# Transcriptional profiling of liver tissues in chicken embryo at day 16 and 20 using RNA sequencing reveals differential antioxidant enzyme activity

**DOI:** 10.1371/journal.pone.0192253

**Published:** 2018-02-06

**Authors:** Shaohua Yang, Lu Lu Wang, Zhaoyuan Shi, Xiaoqian Ou, Wei Wang, Xue Chen, Guoqing Liu

**Affiliations:** 1 College of Food Science and Engineering, Hefei University of Technology, Hefei, Anhui, P. R. China; 2 Agricultural Products Quality and Safety Supervision and Management Bureau, Xuancheng, Anhui, P. R. China; Alexandria University, EGYPT

## Abstract

Considering the high proportion of polyunsaturated fatty acids, the antioxidant defense of chick embryo tissues is vital during the oxidative stress experienced at hatching. In order to better understand the mechanisms of the defense system during chicken embryo development, we detected the activity of antioxidant enzymes during the incubation of chicken embryo. Results showed that the activity of superoxide dismutase (SOD) and (GSH-PX) in livers were higher than those in hearts. Based on these results, liver tissues were used as the follow-up study materials, which were obtained from chicken embryo at day 16 and day 20. Thus, we used RNA sequencing (RNA-Seq) analysis to identify the transcriptome from 6 liver tissues. In total, we obtained 45,552,777–45,462,856 uniquely mapped reads and 18,837 mRNA transcripts, across the 6 liver samples. Among these, 1,154 differentially expressed genes (*p*<0.05, foldchange≥1) were identified between the high and low groups, and 1,069 GO terms were significantly enriched (*p*<0.05). Of these, 10 GO terms were related to active oxygen defense and antioxidant enzyme activity. GO enrichment and KEGG pathway analysis indicated that *GSTA2*, *GSTA4*, *MGST1*, *GPX3*, and *HAO2* participated in glutathione metabolism, and were considered as the most promising candidate genes affecting the antioxidant enzyme activity of chicken embryo at day 16 and day 20. Using RNA-Seq and differential gene expression, our study here investigated the complexity of the liver transcriptome in chick embryos and analyzed the key genes associated with the antioxidant enzyme.

## Introduction

Oxidative stress is always common in poultry production. During embryo growth, more oxygen is required to provide energy. However, elevated oxygen concentrations lead to high levels of reactive oxygen species (ROS) [1], which may which may cause protein and lipid oxidation[2]. Therefore, ROS has been proposed to cause many diseases and pathological changes during chick embryo development [3], especially in the cardiovascular system. Wells showed that excess ROS could have a teratogenic effect on developing embryos[4] as well as induce neural tube defects[5]. Meanwhile, it was found that ROS can also cause myocardial hypertrophy in the developing chick embryo [6]. The damaging effects of ROS can be exerted on the developing embryos in a directly or indirect style. Thus, antioxidant defences play a very important role in chick embryonic development.

In fact, the integrated antioxidant systems within the egg and embryonic tissues are crucial for the protection of chick embryo in its development. Of these, the main antioxidant enzymes, superoxide dismutases (SOD), glutathione peroxidase (GSH-PX), glutathione S transferases (GSTs), Peroxidase (POD) and catalase(CAT), can clearly serve as a major defense line against ROS during the oxidative stress experienced at hatching in chick embryo[7]. During the incubation period, SOD converts highly reactive superoxide anions into H_2_O_2_ and O_2_, Catalase (CAT) catalyzes the dismutation of H_2_O_2_ to form the neutral products O_2_ and H_2_O, and GSH-PX catalyzes the reductive destruction of hydrogen and lipid hydroperoxides with glutathione as an electron donor [8]. Meanwhile, the end product of SOD is decomposed then to scavenge ROS [9]. Additionally, GSH-PX degrades hydrogen peroxide and other peroxides.

The activities of these antioxidant enzymes are close relevant to higher levels of environmental oxygen. Using the chick embryo, van Golde et al[10] investigated the relations between hyperoxia and antioxidant enzyme activity, and found that SOD activity had a 2- to 10-foldincrease and Catalase and GPx enzyme activities remain almost the same in heart, liver, intestine and lungs during incubation at different time points. Starrs et al [11] examined the activities of catalase, SOD and GPx in the developing lungs of the chicken and showed that SOD activity decreased, whereas catalase and GPx activities were significantly increased in late incubation. Nevertheless, Dhage et al [12] revealed there was a significant increase of the SOD activity in the chick embryo from Day 4–11 (units/mg protein), respectively. Considering these inconsistent results, we still do not know the mechanism of genes that regulate antioxidant activity (SOD, CAT, and GSH-PX) during embryo development. Therefore, clarification of the differential gene expression underlying antioxidant enzyme activity in embryo development will have both biological and economic significance.

As a research method, RNA-Seq provides a comprehensive and accurate tool for gene expression pattern analysis [13]. Among these, RNA-Seq results show gene structure, gene biological function, and new transcripts[14–15]. There have been several studies on various types of transcriptomes using RNA-Seq techniques, such as on fish [16–17], mice[18], cows[19], and pigs [20]. However, no studies on the chicken embryo transcriptome by RNA-Seq have been published. Thus, for a better understanding of the adaptive mechanism of antioxidant enzyme activity during the oxidative stress experienced at hatching in embryos, we examined liver transcriptome data from different incubation days in order to determine the key genes that were associated with antioxidant enzyme activity.

## Materials and methods

### Ethics statement

All procedures for animal handling were reviewed and approved by the Institutional Animal Care and Use Committee (IACUC) of Hefei University of Technology (Permit Number: DK838).

### Materials

Fertilized eggs were purchased from a hatchery (Changlv Native Products, Nanjing, China), and were incubated at 37.8°C and 60% relative humidity in an incubator (Photoshop Solar Energy Co., Zibo, China). Fertilized eggs were studied at day 14, 15, 16, 17, 18, 19, and 20 of incubation. SOD, GSH-PX, Peroxidase (POD) and total protein quantitative assay kits were provided by Jiancheng Bioengineering Institute (Nanjing, Jiangsu, China).

### Preparation of tissues

Fertilized eggs at day 14, 15, 16, 17, 18, 19, and 20 of incubation were obtained, peeled, and the heart and liver tissues were removed, respectively. The removed tissues were rinsed with cold saline (0.86%) to remove the blood, immediately frozen in liquid nitrogen, and placed in a centrifuge stored in an ultra-low temperature freezer.

### Determination of activity of antioxidant enzymes

The collected tissue samples were weighed and made into a 20% tissue homogenate by adding the appropriate amount of saline (0.86%), according to the weight of the volume, then centrifuged at 4,000 rpm/min for 10 min at 4°C. The supernatant was obtained for further analysis. The activities of SOD, POD and GSH-PX were measured using commercial assay kits purchased from Nanjing Jiancheng Bioengineering Institute (Nanjing, Jiangsu, China). The antioxidant experiment was conducted in triplicate, and results were analyzed using SPSS 16.0 software (SPSS, Inc, Chicago, IL, USA). Comparison between groups was analyzed by One-way analysis of variance (ANOVA) followed by Duncan's multiple range tests and the results were considered statistically significant at P < 0.05.

### RNA isolation and quality assessment

Liver tissues at day16 and day 20 were chosen for transcriptome study. The total RNA was extracted from the embryo tissues using the Trizol method (Invitrogen, Carlsbad, CA) according to the manufacturer’s instructions. RNA degradation and contamination was monitored on 1% agarose gels. Furthermore, the Nanophotometer (IMPLEN, CA, USA) was used to check RNA purity, and RNA concentration was measured using Qubit RNA Assay Kit in Qubit 2.0 Fluorometer (Life Technologies, CA, USA). The RNA integrity was assessed with the RNA Nano 6000 Assay Kit of the Bioanalyzer 2100 system (Agilent Technologies, CA, USA).

### Library construction and RNA sequencing

A total amount of 3 μg RNA per sample was used as input material for the RNA sample preparations. Sequencing libraries were processed using NEBNext® Ultra™ RNA Library Prep Kit for Illumina® (NEB, USA), following the manufacturer’s recommendations. Cluster generation of the index-coded samples was conducted on a cBot Cluster Generation System using Tru Seq PE Cluster Kit v3-c Bot-HS (Illumia) according to the manufacturer’s instructions. After cluster generation, the library preparations were sequenced on an Illumina Hiseq platform, and 125-bp/150-bp paired-end reads were generated.

### Quality control

Raw data (raw reads) of Fastq format were first processed through in-house perl scripts. In this step, clean data (clean reads) were acquired by removing reads containing adapter, reads containing ploy-N, and low quality reads from raw data. At the same time, Q20 (the proportion of bases with a phredbase quality score greater than 20; i.e., the proportion of read bases whose error rate is less than 1%), Q30 (the proportion of bases with a phredbase quality score greater than 30; i.e., the proportion of read bases whose error rate is less than 1%), and GC content of the clean data were calculated. All the downstream analyses were based on the clean data.

### Reads mapping

Reference genome and gene model annotation files were downloaded from a genome website directly (ftp://ftp.ensembl.org/pub/release-83/gtf/gallus_gallus/). Index of the reference genome was established using Bowtie v2.2.3. Because TopHat can generate a database of splice junctions based on the gene model annotation file (which possesses better mapping results than other non-splice mapping tools), paired-end clean reads were aligned to the reference genome using TopHat v2.0.12.

### Quantification of gene expression level

The number of reads mapped to each gene was calculated by HTSeq v0.6.1. FPKM, expected number of Fragments Per Kilobase of transcript sequence per Millions base pairs sequenced, is currently the most commonly used method for estimating gene expression levels. FPKM of each gene was calculated based on the length of the gene and reads count mapped to this gene.

### Differential expression analysis

Differential expression analysis was conducted using the DESeq R package (1.18.0). DESeq facilitates accurate comparisons between antioxidant enzyme activity of liver tissues by normalizing the number of reads, and provides statistical routines for determining differential expression in digital gene expression date using a model based on the negative binomial distribution. The resulting *P*-values were adjusted using the Benjamini and Hochberg’s approach for controlling the false discovery rate. Genes with an adjusted *P*-value <0.05 and log_2_ (Fold change) ≥1 found by DESeq were assigned as differentially expressed.

### GO and KEGG enrichment analysis of differentially expressed genes

Gene Ontology (GO) enrichment analysis of differentially expressed genes was performed by the GOseq R package, in which gene length bias was corrected. GO terms with *P*< 0.05 were considered significantly enriched by differentially expressed genes.

KEGG provides comprehensive database resources for research of high-level functions and utilities of biological systems (http://www.genome.jp/kegg/). Statistical enrichment of differential expression genes in KEGG pathways was evaluated by KOBAS software.

### Real-time quantitative reverse-transcription-PCR (qRT-PCR)

To verify the accuracy and repeatability of the transcription sequencing results, 10 differentially expressed genes were randomly selected to be detected using qRT-PCR. Designed by Primer3 (http://fokker.wi.mit.edu/primer3/input.htm), the primer sequences were shown in S1 File. The housekeeping gene *GAPDH* was used to correct the mRNA levels of differentially expressed genes. qRT-PCR was carried out in triplicate with the LightCycler ® 480 SYBR Green I Master Kit (Roche Applied Science, Penzberg, Germany), in a 15 μ L reaction on a LightCycler480 (Roche), using the following program: 95°C for 8 min, 45 cycles of 95°C for 10 s, 60°C for 15 s, 72°C for 10 s, and 72°C for 10 min.

## Results

### Antioxidant enzyme activities of embryos

**Fig 1:**
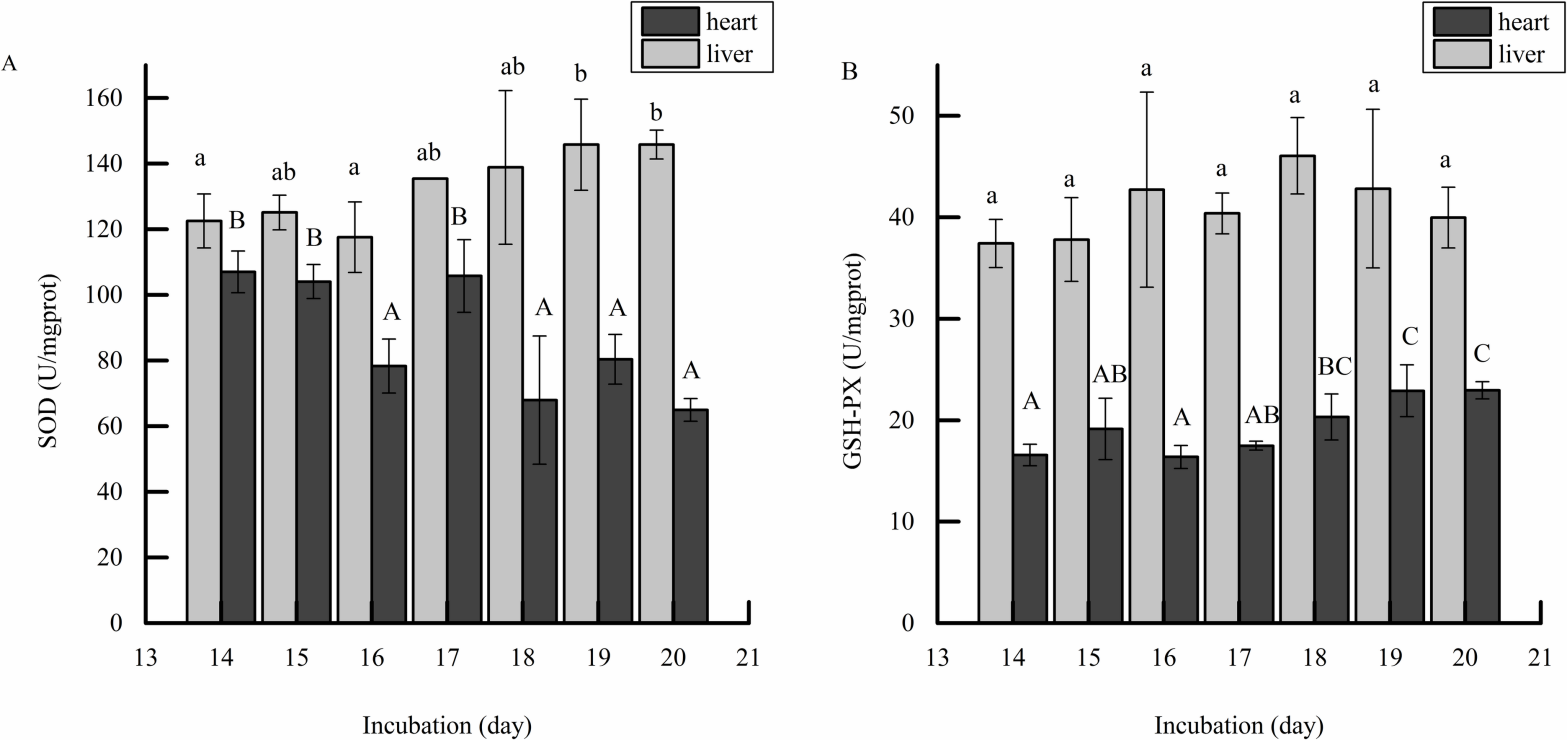
Effect of incubation day on SOD and GSH-PX activity in embryonic liver and heart. **Note:** The SOD activity (A) and GSH-PX (B) of heart and liver tissues were determined at days 14 to 20. The value of each fraction was the mean± standard deviation (n = 3), different letters (a, b) above columns indicate significant differences (p<0.05) in liver tissues, different letters (A, B) above columns indicate significant differences (p<0.05) in heart tissues.

**Fig 2:**
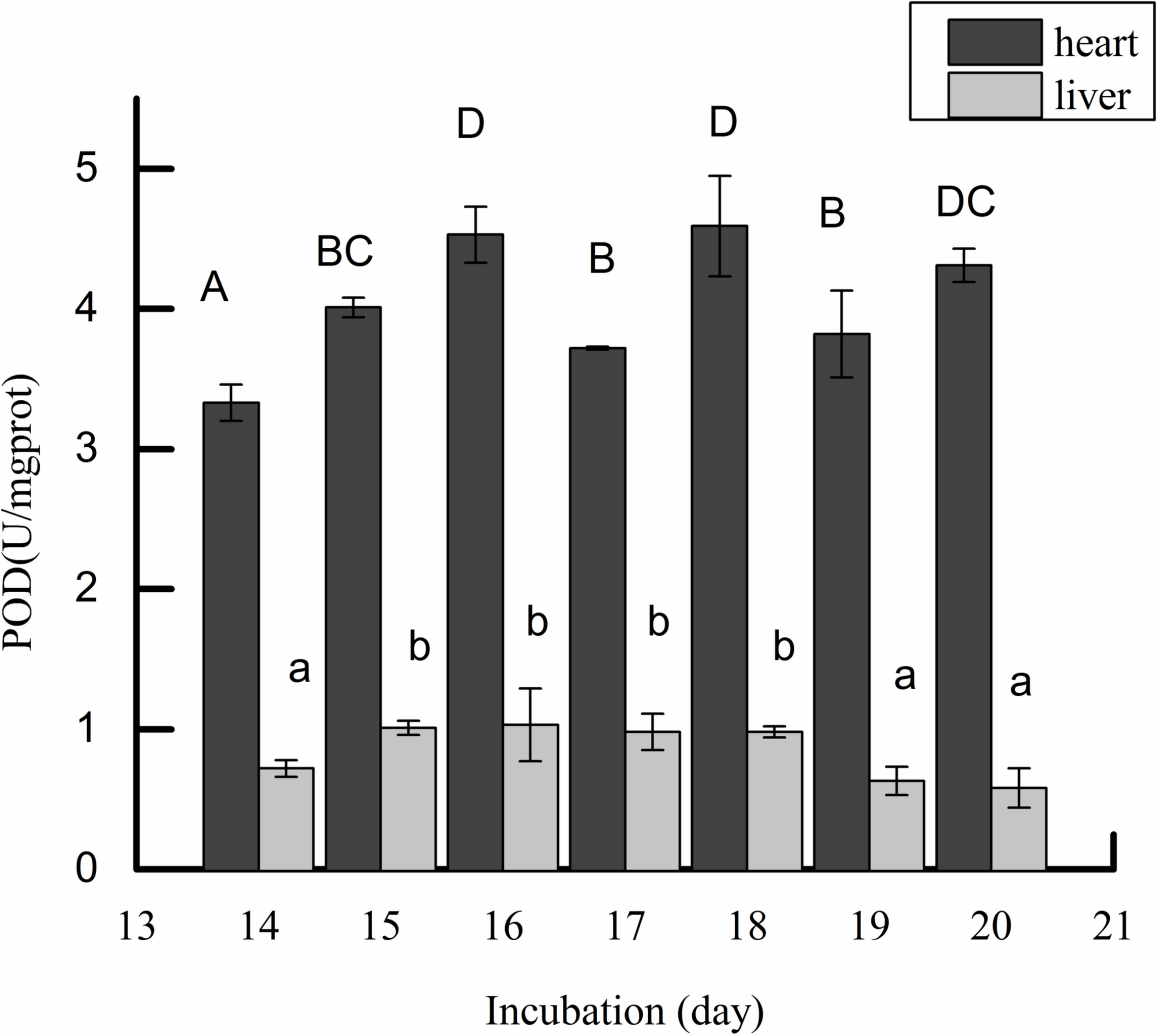
Effect of incubation day on POD activity in embryonic liver and heart. **Note:** The POD activity of heart and liver tissues was determined at days 14 to 20. The value of each fraction was the mean± standard deviation (n = 3), different letters (a, b) above columns indicate significant differences (p<0.05) in liver tissues, different letters (A, B) above columns indicate significant differences (p<0.05) in heart tissues.

Antioxidant enzyme activities of SOD, GSH-Px and POD were detected in the incubation period. As shown in Fig 1A, the activity of SOD in the liver was higher than that in the heart tissue during incubation at different time points. In addition, there was a slight increase in SOD activity in the liver, while it decreased in the heart with the increase in the incubation period. Fig 1A showed that SOD activity in the liver had a significant change from days 14 to 20 (25.86% increase, P<0.05) while Fig 1B showed that GSH-PX activity in the heart had an extremely significant change from days 14 to 20 (43.75% increase, P<0.01). Overall, the activity of GSH-PX in the liver tissue was significantly higher than in that in the heart. Meanwhile, the activity of POD in the heart and liver is shown in Fig 2. The results showed that POD activity in the liver increased initially and then decreased, and its activity had a significant change from days 14 to 20 (28.69% decrease, P<0.05). In the heart, the activity of POD increased steadily. According to the antioxidant activities, liver tissues at day 16 and 20 were used for further study.

### Sequencing and mapping of the liver transcriptome

In total, we obtained 51,868,410–58,937,096 paired-end reads per sample. After removing the sequencing adaptors and poor quality reads, we acquired a total of 336,724,356 clean reads, and the total read length was 50.5 gigabases (GB) for six samples **(**Table 1**)**. The data sets analyzed are available in the NCBI GenBank (https://www.ncbi.nlm.nih.gov/genbank) and the BioProject ID is PRJNA416967 (SRP123539). In this study, the Pearson correlation method was used for the calculation of the correlation coefficient (R^2^). The result showed that R^2^of biologically repeated samples was higher than 0.92, indicating that the similarity of the three biological replicates within each group was sufficiently high.

**Table 1:** The basic statistics for RNA-Seq reads of 6 embryos with different antioxidant enzyme activity.

Sample name	Raw reads	Clean reads	clean bases	Error rate (%)	Q20 (%)	Q30 (%)	GC content (%)
Day20-1	63728726	57423736	8.61G	0.02	97.38	92.63	49.13
Day20-2	58623074	52732394	7.91G	0.02	97.37	92.6	49.39
Day20-3	65547680	58937096	8.84G	0.02	97.41	92.71	49.3
Day16-1	57292698	51868410	7.78G	0.02	97.52	92.95	49.37
Day16-2	64924706	58410598	8.76G	0.02	97.43	92.79	49.63
Day16-3	63551732	57352122	8.6G	0.02	97.42	92.73	49.59

**Note:** Day 20 means the liver tissues in chicken embryo at day 20 and Day 20–1, Day 20–2, Day 20–3 mean the three biological replicate liver tissues in Day20 group, the rest of groups share the same name rules. Q20, the proportion of bases with a phred base quality score greater than 20; i.e., the proportion of read bases whose error rate is less than 1%.Q30, the proportion of bases with a phred base quality score greater than 30; i.e., the proportion of read bases whose error rate is less than 0.1%.

### Different gene expression between high and low groups for SOD activity

**Fig 3:**
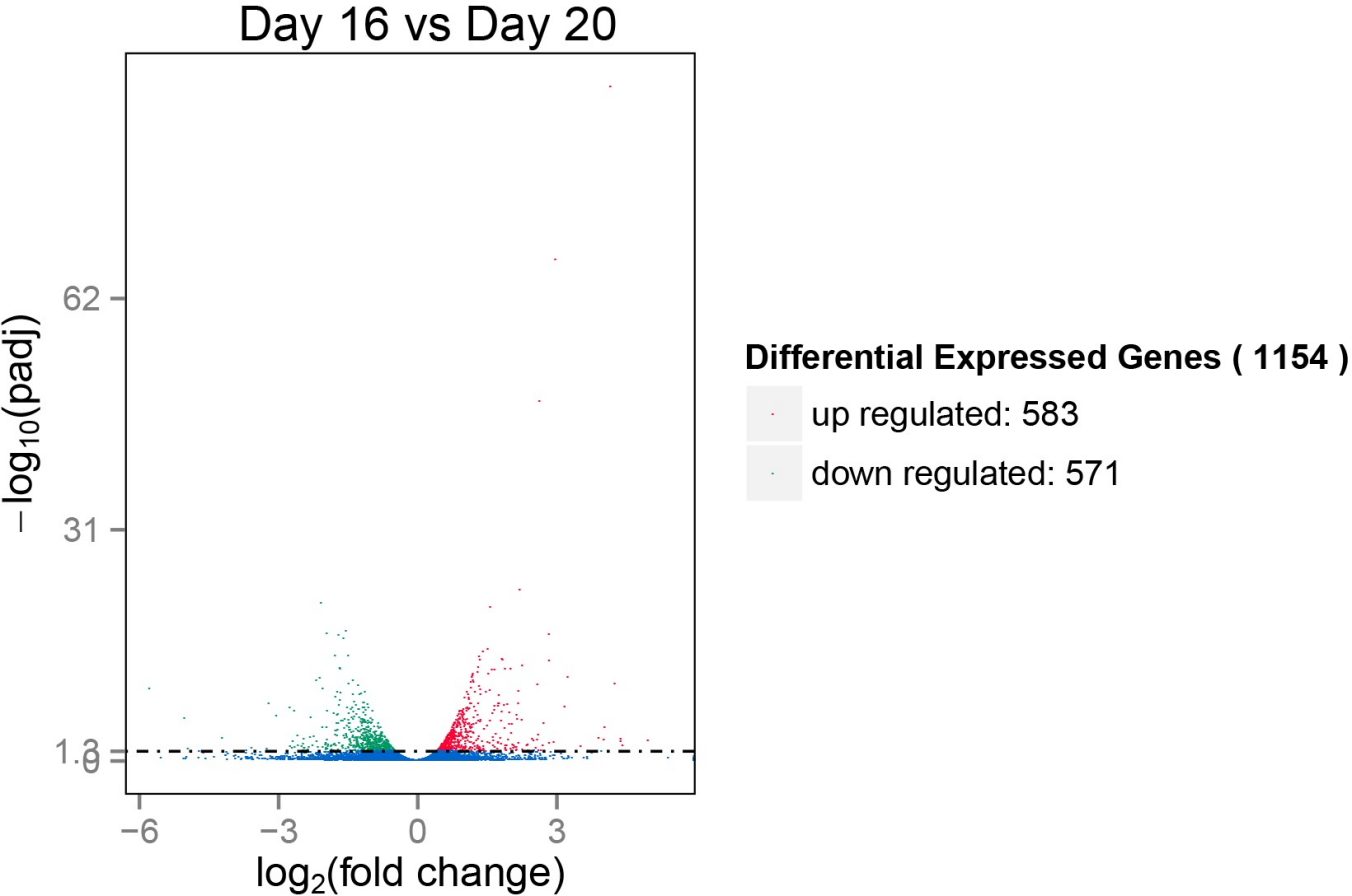
Volcano plot displaying DEGs within two different comparison groups. Note: the y-axis shows the mean expression value of log_10_(q-value), and the x-axis displays the log_2_fold change value. The blue dots represent the transcripts that did not reach statistical significance (q > 0.05); the red (up-regulated) and green dots (down-regulated) represent those whose expression levels were significantly different (q < 0.05); the blue dots represent the transcripts did not reach statistical significance (q > 0.05).

Using the DEseq R package, the differential gene expression profile between liver tissues in chicken embryo at day 16 and day 20 was examined. In total, 18,837 genes were plotted. Meanwhile, 1,154 (571 down and 583 up) differentially expressed genes were identified (DEGs) at an FDR (false discovery rate) adjusted p-value <0.05, and absolute value of fold change ≥1 in two samples. Volcano plots of the two comparison groups that are differentially expressed illustrate the distinct transcriptional profiles, displayed in Fig 3. The details of all DEGs are shown in S2 File.

### GO and pathway analysis of the DGEs

To further evaluate the function of differentially expressed genes, GO classification analysis was used to annotate all genes identified from liver tissues, and formed into three categories: cellular components, biological processes, and molecular functions. These 1154 differential expressed genes were analyzed by GO enrichment, and 7731 GO terms were obtained. Of these, 1069 GO terms (13.83%) were significantly enriched (*p*<0.05) (S3 File). GO terms with P-value less than 0.05 were considered significantly enriched by DEGs. Among these GO terms, there are some GO terms related with cell cycle process, regulation of cell cycle and response to stress etc. In addition, there are 10 GO terms significantly related with antioxidant enzyme activity (P<0.01), such as glutathione transferase activity, a response to reactive oxygen species (Table 2). Meanwhile, we conducted metabolic pathway analysis using KOBAS software; the details of the significant pathway in the two-comparison group are shown in S4 File. The results of the KEGG analysis showed that several important pathways, such as “glyoxylate and dicarboxylate metabolism”, “carbon metabolism”, and the “p53 signaling pathway” were significant.

**Table 2:** Summary of the GO analysis of antioxidant enzyme activity-related changes.

GO ID	GO term	term type	Total No. of DEGs	No. of DEGs	P-value
GO:0000302	response to reactive oxygen species	biological process	90	15	0.00203
GO:0004364	glutathione transferase activity	molecular function	10	4	0.00207
GO:0006979	response to oxidative stress	biological process	195	26	0.00232
GO:0016559	peroxisome fission	biological process	9	4	0.00336
GO:0006982	response to lipid hydroperoxide	biological process	2	2	0.00380
GO:0034599	cellular response to oxidative stress	biological process	113	17	0.00413
GO:0007031	peroxisome organization	biological process	28	6	0.01482
GO:0004602	glutathione peroxidase activity	molecular function	11	3	0.02426
GO:0033194	response to hydroperoxide	biological process	10	3	0.03135
GO:0034614	cellular response to reactive oxygen species	biological process	61	9	0.03333

**Note:** GO ID indicates the label information in the Gene Ontology database, GO term refers to the description information of Gene Ontology function, P<0.05 means the function is an enriched item

### Real-time quantitative PCR

**Fig 4:**
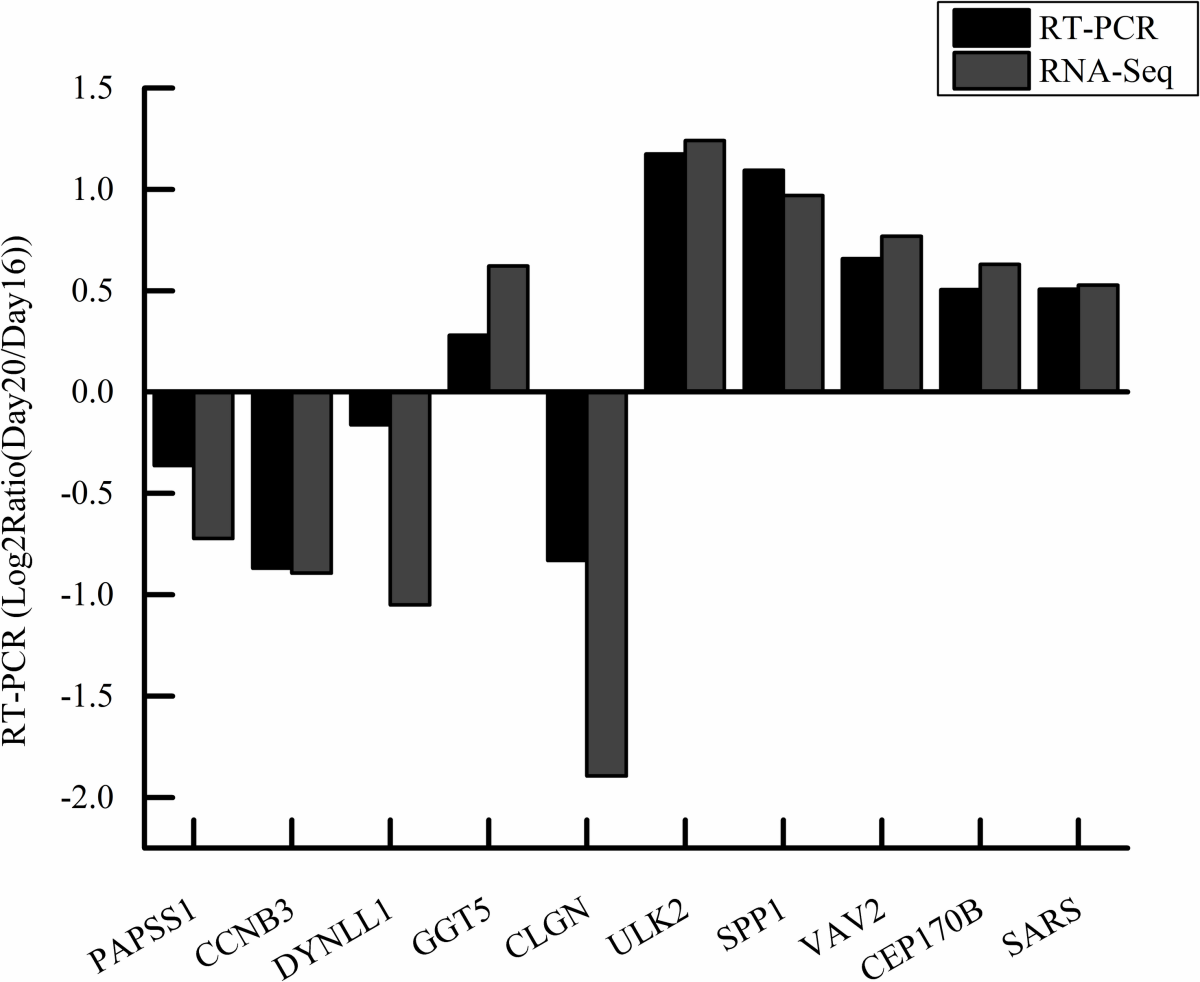
Validation of the gene expression profile by real-time PCR. **Note:** The x-axis represents the gene name, the y-axis represents the log_2_Ratio (Day20/Day16), different color columns represent data from RT-PCR or RNA-Seq.

Ten genes *(PAPSS1*, *CCNB3*, *DYNLL1*, *GGT5*, *CLGN*, *ULK2*, *SPP1*, *VAV2*, *CEP170B*, and *SARS*) were analyzed to confirm expression profiles and validate the transcriptome analysis results (Fig 4). The results showed that the gene expression levels were all consistent with mRNA-Seq results, which confirmed that the results obtained from the transcriptome sequencing platform were accurate.

### Candidate genes

According to the GO database, within significantly enriched biological processes, 8 biological processes were related to reactive oxygen species and antioxidant enzyme activities. Among these processes, the most significant one was “response to reactive oxygen species”. Integrated analysis of DEGs, GO and pathway results, and gene function allow us to suggest *GSTA2*, *MGST1*, *GSTA4*, *GPX3* and *HAO2* (Table 3) as the 5 promising candidate genes for affecting the activity of antioxidant enzymes during chicken embryo development.

**Table 3:** Differentially expressed genes identified from 10 significantly enriched GO terms related to antioxidant enzyme activity.

Symbol	log_2_fold change	Gene name	p-value	Gene function
*APOA4*	1.3571	apolipoprotein A4	7.92E-18	removes cholesterol from peripheral cells and directs it to the liver for metabolism; antioxidant
*GSTA2*	-1.2497	glutathione S-transferase alpha 2	1.66E-13	possesses glutathione-dependent steroid isomerase activity and glutathione-dependent peroxidase activity
*GSTA4*	-1.1847	glutathione S-transferase alpha 4	1.93E-08	glutathione peroxidase activity and play effect on the detoxification of lipid peroxidation products
*LTC4S*	-1.3983	leukotriene C4 synthase	2.70E-06	catalyzes the formation of pro-inflammatory lipid mediator-leukotriene C4
*MGST1*	-0.64393	microsomal glutathione S-transferase	4.40E-05	possesses glutathione S-transferase activity and catalyzes the reduction of certain lipid hydroperoxides
*PEX11A*	-0.83865	peroxisomal biogenesis factor 11 alpha	0.000914	peroxisome abundance and fatty acid metabolism
*GPX3*	0.48585	glutathione peroxidase 3	0.002527	catalyzes the reduction of organic hydroperoxides and protects cells against oxidative damage

## Discussion

RNA-Seq has been applicated in many fields in chickens. Monson [21] established the liver library of domesticated turkey and wild turkey with AFB1 treatment by RNA-Seq and obtained 89.2Gb of sequence. Sporer [22] utilized the microarray to detect the key genes in turkey skeletal muscle development and identified DEGs between two genetic lines of turkeys. Zhuo [23] revealed the differences of gene expression in the abdominal fat with high and low feed efficiency commercial broiler chickens. RNA-Seq was first used for analysis of early embryonic development in cattle and provided a method for further study mammalian embryonic development [24]. Based on those studies, we evaluated the whole genome transcriptome profile of chicken embryo liver tissues on different incubation days using RNA-Seq, with the aim of determining key genes that regulate the activity of antioxidant enzymes in this study.

Compared with mammals, chicken embryonic development is carried out in a semi-closed system, and these natural antioxidants (SOD, GSH-PX and CAT) have been suggested to play a central role at hatching [25]. Therefore, the aim of this study is to detect the differentially expressed genes associated with the activities of antioxidant enzymes during chick embryonic development. In the present study, the activity of SOD and GSH-PX was significantly higher in the liver than in the heart. In the heart, GSH-PX activity value was half of that in the liver, which was consistent with results shown by Surai [26]. Additionally, CAT in the liver might be less important in ROS defense[27]. Thus, the results of antioxidant enzyme activity revealed that the liver was an important source of antioxidants. The different activities of antioxidant enzymes in the liver may be caused by different regulation of gene expression. According to the antioxidant enzyme activity detected in this study, the cDNA library was established by the liver. The transcript was deeply sequenced and the package of DESeq and Cuffdiff was used for analyses. We obtained 1,154 differentially expressed genes, while some of the genes have a known function, e.g., *GSTA*, *GGT*, *GPX3*, studies have also reported that these genes are associated with antioxidant enzyme activity [28–30]. Of these, DEGs, *APOA4*, *LTC4S*, *GSTA2*, *MGST1*, *GSTA4*, *GPX3*, and *PEX11A* were suggested to be the promising candidate genes for affecting the mechanisms of the defense system in response to ROS during chicken embryo development.

Apolipoprotein (apo) A-IV (*APOA4*) was detected in the processes of “response to reactive oxygen species”, “response to oxidative stress”, “response to lipid hydroperoxide”, “cellular response to oxidative stress”, and “response to hydroperoxide”. *APOA4* is a 46 kDa glycoprotein and encodes a protein consisting of 396 amino acid residues. Kumar et al. [31] reported that *APOA4* is an important mediator of lipid metabolism and has antioxidant activity. Studies have shown that the antioxidant effect of *APOA4* is mediated by its ability to bind to the surface of the abundant lipoprotein lipase within atherosclerotic plaques [32]. Ostoa et al [33] suggested that *APOA4* and *APOA1* accumulate in diseased artery walls to help protect against lipid peroxidation.

As a member of the *MAPEG* (Membrane Associated Proteins in Eicosanoid and Glutathione metabolism) family of transmembrane proteins, leukotrieneC4 synthase (*LTC4S*) was down-regulated in this study. *LTC4S* encodes an enzyme that catalyzes the first step in biosynthesis of cysteinyl leukotrienes (LT), which possess important functions in inflammation [34]. Zhang et al. [35] showed that *LTC4S* and *Orai3* can promote vascular smooth muscle cell migration and neointima formation by changing Akt signaling.

Glutathione S-transferase alpha 2 (*GSTA2*) is a member of a family of glutathione S-transferases (GSTs), located in a cluster of similar genes and pseudogenes on chromosome 6. *GSTA2* plays a role in detoxification by adding glutathione to target electrophilic compounds. Located in a cluster mapped to chromosome 6, *GSTA2* is the most abundantly expressed glutathione S-transferase in the liver [36–37]. Additionally, *GSTA2* has glutathione peroxidase activity, thereby protecting the cells from reactive oxygen species and the products of peroxidation. Tetlow [38] suggested that *GSTA2* is a majorline of defense against oxidative stress. Glutathione S-transferase alpha4 (*GSTA4*) encodes a glutathione S-transferase belonging to the alpha class. The alpha class genes are highly related and encoded enzymes with glutathione peroxidase activity, that have a function in the detoxification of lipid peroxidation products [39]. Shearn [40] suggested that *GSTA4* is a phase 2 detoxifying enzymes, and that its expression increases in response to oxidative stress.

The microsomal glutathione S-transferase 1 (*MGST1*) gene encodes a protein that catalyzes the combination of glutathione to electrophiles and the reduction of lipid hydroperoxides. In addition, it scavenges reactive intermediates through its glutathione dependent transferase and peroxidase activities [41]. Glutathione peroxidase 3 (*GPX3*) belongs to the glutathione peroxidase family, which catalyzes the reduction of organic hydroperoxides and hydrogen peroxide by glutathione, and protects cells against oxidative damage [42]. Olson et al. [43] suggested that *GPX3* was the only known selenocysteine-containing extracellular form of glutathione peroxidase. Barrett et al. [44] reported that knockdown of *GPX3* in the human colon cancer cell line Caco2 caused an increase in ROS production. In addition, Xu et al.[45] suggested that vitamin E improves the antioxidant defense mechanisms, and enriches the *GPX3* mRNA and protein expression levels, thereby enhancing the testicular antioxidant capacity. Peroxisomal biogenesis factor 11 alpha (*PEX11A*) is the richest ingredient of the peroxisomal membrane, and essential for proliferation of peroxisomes [46]. Rodríguez-Serrano et al [47] reported that in *Arabidopsis*, *PEX11A* lines exhibited higher levels of lipid peroxidation content and lower expression of genes involved in antioxidative defense and signaling. Weng et al. [48] found that the deficiency of *PEX11A* is related to peroxisome abundance reduction.

KEGG metabolic pathway was used to analyze the function of differentially expressed genes, and 16 out of 139pathway terms was significantly enriched. Of these, “Glutathione metabolism” and “Glyoxylate and dicarboxylate metabolism” were pathway terms related to ROS defense. As a low molecular weight tripeptide, glutathione (GSH) is only present in a small quantity in the oxidation process[49]. Reduced GSH reduces the peroxide toH_2_O, and free radical reactions in vivo can be maintained. “Glutathione metabolism” pathway involved 9 differentially expressed genes, which were all down-regulated. Presumably, the mechanism is that the up-regulation of GPX3 (1.11.1.9) promotes the conversion of GSH to GSSG, and glutathione reductase (GSR) (1.8.1.7) catalyzes the reduction of GSSG to GSH with NADPH as an H donor. Meanwhile, changes in the differentially expressed genes indicate that the glutathione metabolism in the liver is accelerated during the later stage of hatching. Additionally, hydroxyacid oxidase 2 (*HAO2*) was up-regulated in the conversion process of Glycolate to H_2_O_2_. The up-regulation of *HAO2* indicated that it can catalyze more glycolate and generate more H_2_O_2_. Mattu et al. [50] also revealed that the increased expression of *HAO2* caused increased ROS production and lipid peroxidation.

SOD, GSH-PX, and POD together play a role in the fight against ROS together in the embryo; the SOD activity is decreased with the generation of H_2_O_2_, when scavenging O^2-^. While adding CAT and GSH, H_2_O_2_can be decomposed. In this study, most genes in the “glutathione metabolism” pathway were down-regulated, indicating more GSH involved in scavenging H_2_O_2_, thereby protecting SOD activity. PEX11a down-regulated revealed the decrease in POD activity, and more POD participating in ROS defense with GSH.

In this comprehensive analysis of GO enrichment and the KEGG pathway, we discovered that some different gene expression not only exists in significantly enriched GO terms, but also participates significantly in the KEGG pathway; these genes were *MGST1*, *GSTA2*, *GSTA4*, *GPX3*, *HAO2*. This result revealed that these 5 genes may be key genes affecting ROS defense and antioxidant enzymes. These genes were associated with glutathione metabolism, and thus affected enzyme activity. This discovery also confirmed that GSH-PX played a primary role in antioxidant defense in the liver. Further research is required to understand the molecular mechanisms of these candidate genes on ROS defense and antioxidant enzyme in chickens.

## Conclusions

Chicken embryo liver tissues at day 16 and day 20 were used as the follow-up study materials. In this study, we provided a comprehensive analysis of the complexity of the liver tissue transcriptome, and identified 1,154 differentially expressed genes between different incubation periods, with high and low antioxidant enzyme activity. GO enrichment and pathway analysis revealed 5 key genes affecting ROS defense and antioxidant enzymes, including *MGST1*, *GSTA2*, *GSTA4*, *GPX3* and *HAO2*.

## Supporting information

S1 File PCR primers for qRT-PCR validation of 10 DEGs between the two different comparison groups. (DOCX)

S2 File List of DEGs in liver tissues within two different comparison groups. (XLSX)

S3 File GO enrichment list of DEGs between high and low antioxidant enzyme activity in biological processes. (XLSX)

S4 File List of significant KEGG pathway categories for DEGs. (XLSX)
