# Supplementary material for: Transcriptional profiling of liver tissues in chicken embryo at day 16 and 20 using RNA sequencing reveals differential antioxidant enzyme activity
Source: PLoS One. 2018 Feb 6;13(2):e0192253. doi: 10.1371/journal.pone.0192253 (PMC5800670; doi:10.1371/journal.pone.0192253)
Supplement: S1 File — (DOCX) [file pone.0192253.s001.docx]

**File S1.** **PCR primers for qRT-PCR validation of 10 DEGs between the two different comparison groups**

| **Gene** | **Primer sequences (5'to3')** | **Length（bp）** |
| --- | --- | --- |
| *PAPSS1*-F | CACAGGAAGACAAGGAAAGAC | 200 |
| *PAPSS1*-R | TCCACCTACAAGCCAGTTC |  |
| *CCNB3*-F | GGTGGAGGACATAGACAAGGA | 116 |
| *CCNB3* -R | TGTAATCAGGGAGCAGGAACT |  |
| *DYNLL1*-F | ATCGTGGGAAGGAACTTTG | 89 |
| *DYNLL1*-R | AAGAGGAGAATAGCGACTTGG |  |
| *GGT5*-F | ATGAGGGAGAGATAGGAAAGG | 191 |
| *GGT5*-R | GAGGATGAACAGGAGCACA |  |
| *CLGN*-F | CCTCCAGTAAACCCTCCTAAA | 123 |
| *CLGN*-R | CTCATCTTCATCCCAGTCATC |  |
| *ULK2*-F | GTGAGCATAAGGGACCACA | 72 |
| *ULK2*-R | GGCAGAACAAGGACAACAA |  |
| *SPP1*-F | CTGGCATTTCTTTGCTTGTG | 111 |
| *SPP1*-R | GCTCCTGGGGTCGTATTTT |  |
| *VAV2*-F | AGCAGGGCTTCCAGTTCTT | 57 |
| *VAV2*1-R | GCTCCATCCACTTCCTCTTC |  |
| *CEP170B*-F | TAGTTATCGCACCCCTCTTT | 90 |
| *CEP170B*-R | CCTTCTGTCCTCCTTGTTGT |  |
| *SARS* -F | CTCGTGTCCTGCTCCAACT | 88 |
| *SARS* -R | CCACCTTGTCCATCATCTTCT |  |
| GAPDH-F | GGAGAAACCAGCCAAGTATG | 142 |
| GAPDH-R | ATCAAAGGTGGAGGAATGG |  |
